# Whether cognitive behavioral therapy is effective for Alzheimer's disease: A protocol for systematic review and network meta-analysis

**DOI:** 10.1097/MD.0000000000023945

**Published:** 2021-01-08

**Authors:** Wan-Qiang Chen, Fang-Fang Wu, Hong-Bo Lv, Wen-Ting Xing, Qi Liu, Jun-Ping Liu, Yong-Gui Ge, Ya-Qin Lu

**Affiliations:** a Department of Rehabilitation, The First Hospital of Lanzhou University; b Evidence-Based Medicine Center, School of Basic Medical Sciences, Lanzhou University, Lanzhou City, Gansu Province; c WHO Collaborating Center for Guideline Implementation and Knowledge Translation; d Department of Sleep, The Third Peoples Hospital of Lan Zhou, Lanzhou, Gansu, China.

**Keywords:** alzheimer's disease, cognitive behavioral therapy, randomized controlled trials

## Abstract

**Background:**

Alzheimer disease (AD) is a progressive neurodegenerative disease characterized by impaired memory and cognitive judgment. It is the leading cause of dementia in the elderly, and its high morbidity and mortality have also brought a significant social burden. So far, there is no method can completely cure Alzheimer's dementia, but there are many non-drug treatments that have been praised by people, especially the cognitive behavioral therapy proposed in recent years. The main purpose of this article is to evaluate the effect of cognitive behavioral therapy on the cognitive function improvement of patients with Alzheimer's dementia.

**Methods:**

We did a network meta-analysis to identify both direct and indirect evidence in relevant studies. A systematic literature search will be performed in the Cochrane Library, PubMed, and EMBASE from inception to October 2020. We extracted the relevant information from these trials with a predefined data extraction sheet and assessed the risk of bias with the Cochrane risk of bias tool.

The outcomes investigated were Mini–Mental State Examination and AD Assessment Scale-Cognitive section scores. We did a pair-wise meta-analysis using the fixed-effects model and then did a random-effects network meta-analysis within a Bayesian framework. The = the Assessment of Multiple Systematic Reviews-2 scale, Preferred Reporting Items for Systematic Reviews and Meta-Analyses scale and Grading of Recommendations Assessment, Development and Evaluation were used to assess the quality and evidence grade of the literature. General characteristics of the eligible randomized controlled trials will be summarized and described. Meanwhile, The ADDIS software will be used to perform the network meta-analysis, and the result figures will be generated by STATA 15.0 software.

**Results:**

Using the draft search strategy of databases and after screening,7 randomized controlled trials met the a priori criteria and were included. This network mate-analysis will be published in a peer-reviewed journal.

**Conclusion:**

Our study will provide evidence for cognitive behavioral intervention in AD patients. And provide recommendations and guidelines for the clinic.

**Protocol Registration:**

INPLASY2020110052.

## 1 Introduction

Alzheimer disease (AD), characterized by cognitive deterioration, behavioral disturbances and even declining activities, is a progressive neurodegenerative disease.^[1]^ The prevalence rate of dementia doubles approximately every 6 years from the age of 65 years, reaching 7% in those aged 75 to 79 years, 12% in those aged 80 to 84 years, 20% in those aged 85 to 89 years, and 40% in those aged 90 years or over.^[2]^ AD may be identified as the global public health issue in the following seasons, with more than 20 million individuals affected all over the world and expected 135 million patients by 2050.^[3]^

Cognitive impairments are AD core clinical symptoms, and they impose the greatest burden on patients and their caregivers. Improving patients’ cognitive function can delay hospitalization, and therefore reduce the costs of national healthcare and improve both patients’ and caregivers’ well-being. ^[4]^ Pharmacological interventions attempting to counteract the lesions have yet to achieve permanent successful results.^[5–7]^

The effect of cognitive behavioral therapy does not lack completely strong evidence, such as a large number of randomized controlled trials (RCTs). A review published in 2019 analyzed data from 33 cognitive therapy studies from 12 countries/regions, including a total of approximately 2000 participants. And it pointed out that, compared with receiving usual treatment or engaging in non-specific activities, people completing cognitive therapy may show some benefits in overall cognition, as well as in more specific cognitive abilities such as verbal fluency, and that improvements may last for at least a few months.^[8]^

NMA is an extension of traditional metaanalysis, which can compare the efficacy of 3 or more interventions at the same time.^[9]^ It allows comparisons of more than 2 interventions in a single, coherent analysis of all the relevant RCTs, when multiple studies are available, it can also be used to combine multiple therapeutic effects and obtain an overall estimate of the effects in the target population. The main advantage is that it can quantitatively compare different measures for the treatment of similar diseases, and sort according to the plan effect of a certain result index, and then choose the best treatment.^[10,11]^

As a consequence, we performed a network meta-analysis of available RCT to review the quantity and quality of research evidence as well as to evaluate the effect of cognitive behavioral therapy on AD.

## 2 Method

### 2.1 Study design

We will conduct a network meta-analysis of RCT on cognitive behavioral therapy for Alzheimer's disease in the past.

### 2.2 Study registration

Ethics approval is not required for this overview of systematic reviews. We will follow the Preferred Reporting Items for Systematic Reviews and Meta-Analyses Protocols checklist for reporting our overview.^[12]^ The study protocol has been registered with the International Platform of Registered Systematic Review and Meta-analysis Protocols (INPLASY) database (protocol number: INPLASY2020110052, DOI:10.37766/inplasy2020.11.0052).

### 2.3 Data sources and search strategy

We followed the guidelines from the Cochrane Diagnostic Test Accuracy Working Group for undertaking and reporting this systematic review. We searched the Cochrane Library, PubMed and EMBASE (between January 1983 and October 2020). Reference lists of articles, grey literature, and conference proceedings will also be searched. The search strategy has been adapted to each database, the search terms include “ Alzheimer's Disease,” “Alzheimer diseasesenile dementia,” “Cognitive Behavioral Therapy,” “Cognitive behavioral intervention,” “Randomized Controlled Trial,” and others. Languages of the publications will be limited to English. The articles were initially identified based on their title. Abstracts of all the identified studies were examined and full papers obtained on potentially eligible studies. References and bibliographies from retrieved articles were also manually examined. A draft search strategy using the Cochrane Library is presented in Table 1, whereas a draft search strategy using PubMed is presented in Table 2.

**Table 1:** A draft search strategy using the Corchrane Library.

#1	MeSH descriptor: [Alzheimer Disease] explode all trees
#2	(Senile Dementia):ti,ab,kw OR (Alzheimer Type Dementia):ti,ab,kw OR (Alzheimer Type Senile):ti,ab,kw OR (Alzheimer Dementia):ti,ab,kw OR (Alzheimer Syndrome):ti,ab,kw (Word variations have been searched)
#3	(AD):ti,ab,kw (Word variations have been searched)
#4	#1 OR #2 OR #3
#5	MeSH descriptor: [Cognitive Behavioral Therapy] explode all trees
#6	(Cognitive Behavior Therap∗):ti,ab,kw OR (Cognit∗ Therap∗):ti,ab,kw OR (Cognitive behavioral intervention):ti,ab,kw OR (cognitive behavioral intervention therapy):ti,ab,kw OR (CBT):ti,ab,kw (Word variations have been searched)
#7	#5 OR #6
#8	MeSH descriptor: [Randomized Controlled Trial] explode all trees
#9	(RCT):ti,ab,kw OR (randomized clinical trial):ti,ab,kw (Word variations have been searched)
#10	#8 OR #9
#11	#4 AND #7 AND #10

**Table 2:** A draft search strategy using PubMed.

#1	“Alzheimer Disease”[Mesh]
#2	“senile dementia”[Title/Abstract] OR “alzheimer dementia”[Title/Abstract] OR “alzheimer type dementia”[Title/Abstract] OR “alzheimer type dementia”[Title/Abstract] OR “AD”[Title/Abstract] OR “alzheimer syndrome”[Title/Abstract]
#3	#1 OR #2
#4	“Cognitive Behavioral Therapy”[Mesh]
#5	“cognitive behavior therap∗”[Title/Abstract] OR (“cognit∗”[All Fields] AND “therap∗”[Title/Abstract]) OR “cognitive behavioral intervention”[Title/Abstract] OR ((“cognition”[MeSH Terms] OR “cognition”[All Fields] OR “cognitions”[All Fields] OR “Cognitive”[All Fields] OR “cognitively”[All Fields] OR “cognitives”[All Fields]) AND “behavioral intervention therapy”[Title/Abstract]) OR “CBT”[Title/Abstract]
#6	#4 OR #5
#7	“Randomized Controlled Trial” [Publication Type] OR “Randomized Controlled Trials as Topic”[Mesh]
#8	“RCT”[Title/Abstract] OR “randomized clinical trial”[Title/Abstract]
#9	#7 OR #8
#10	#3 AND #6 AND #9

### 2.4 Study selection

#### 2.4.1 Type of study

RCTs that explored cognitive behavioral therapy for Alzheimer's disease will be included.

#### 2.4.2 Inclusion criteria

(1) Patients: Elderly people with Alzheimer's disease. There were no restrictions on age, gender or race;
(2) Intervention: Placebo; CS; AMT; NE; usual standard clinical care; CD; PT and CTRL;
(3) Comparator: Cognitive behavioral therapy;
(4) Outcome: Primary outcome measures
  a) mini-mental state examination and AD Assessment Scale-Cognitive section scores served as dependent measures.^[13]^ The mini-mental state examination score is widely used as a parameter to identify a clinically significant decline in cognitive function in AD.^[14]^ The Alzheimer Disease Assessment Scale-Cognitive section consists of 11 tasks measuring the disturbances of memory, language, praxis, attention, and other cognitive abilities which are often referred to as the core symptoms of AD.
  b) Quality of life is measured using the Quality of Life--Alzheimer's disease Scale.^[15]^ The QOL-AD covers 13 domains of quality of life. It has good internal consistency, validity and reliability and its use is recommended by the European consensus on outcome measures for psychosocial interventions in dementia.^[16]^ The Beck Depression Inventory (BDI),^[17]^ State Trait Anxiety Inventory (STAI Y-1, STAI Y-2)^[18]^ and Lubben Social Network Scale (LSNS)^[19]^ assessed anxiety, depression, and social relationships. Higher scores indicated worse anxiety and depression or more frequent social relationships.^[20]^
(5) Studies that their full text was available.

#### 2.4.3 Exclusion criteria

(1) Literatures published repeatedly by the same author or with duplicate data;
(2) AD with other organic diseases;
(3) The protocols are excluded;
(4) Language is not English.

### 2.5 Data extraction

Data were abstracted by 2 investigators. Discrepancies were resolved by a third investigator. Information abstracted included the study population characteristics, Interventions in the experimental group and the control group, the potential risk of bias, and the main results and conclusions of the study.^[21]^

### 2.6 Quality assessment

Two reviewers independently assessed each included RCT by using the Assessment of Multiple Systematic Reviews-2 measurement tool and the statement, for rigorous methodological quality and reporting quality.^[22,23]^ The Assessment of Multiple Systematic Reviews approach consists of 11 items and is featured by good content validity, wide acceptance, recognized reliability, and reproducibility.^[24]^ The ADDIS software and STATA 15.0 were used to analyses data.

Grade evaluation the effect sizes for continuous outcomes were the mean difference (MD). Consistency and inconsistency were the 2 models used to estimate the effect size in ADDIS. A consistency assessment drew conclusions on the effect sizes of the included interventions and estimated the ranking probabilities for all the interventions. The consistency test was judged by node-splitting analysis and an inconsistency model. When the p-value of the node-splitting analysis was greater than 0.05, a consistency mode was selected.^[25]^ Otherwise, an inconsistency model was used. Potential scale reduction factor (PSRF) was used to evaluate the convergence of the model. The closer the PSRF value was to 1, the better the convergence. The convergence of the model was still acceptable if the PSRF value was less than 1.2.

For each intervention, we estimated the ranking probabilities for each treatment at each possible rank. We ran pair-wise meta-analyses to compare the compliance of different non-drug therapies. Bayesian probabilistic networks (BNs) yield a way to construct expert systems by utilizing likelihood as an estimation of unpredictability.^[26]^ We did a pair-wise meta-analysis using the fixed-effects model and then did a random-effects network meta-analysis within a Bayesian framework.

The odds ratio was calculated for dichotomous outcomes (compliance), with 95% credible intervals. We assessed statistical heterogeneity in the pair-wise comparison with an *I*^2^ statistic and the *P*-value.^[27]^ We will use the Grading of Recommendations Assessment, Development and Evaluation framework to assess the quality of evidence, so as to provide strong evidence for the treatment of AD patients and provide recommendations for clinical practice or guidelines. These 5 considerations (study limitations, consistency of effect, imprecision, indirectness, and publication bias) will be applied to assess the quality of evidence.^[28,29]^ It is categorized into 4 levels: high level, moderate level, low level, and very low level.

Meanwhile, we assessed related conflicts of interest. Conflicts of interest-personal, organizational, and financial factors, which may affect the objectivity and independence of guideline contributors are a potential source of bias in the development of clinical practice guidelines.^[30]^

## 3 Result

### 3.1 Study identification and selection

**Figure 1:**
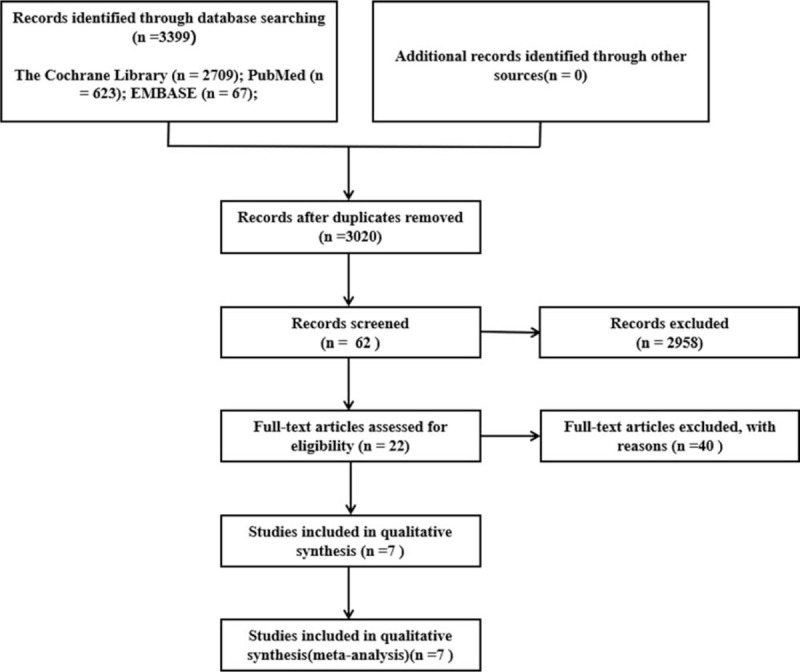
The flowchart of the screening process.

We searched 3399 articles related to Cognitive Behavioral Therapy and AD published from January 1983 to October 22, 2020. By removing duplication and excluding irrelevant articles, after removing duplicates and unrelated articles, 7 articles describing 7 RCTs including 335 patients were eligible for further quantitative analyses. A flow chart of the specific screening procedures is shown in Figure 1.

### 3.2 General characteristics of included studies

We extracted the basic characteristics of some of the included studies. We included 7 RCTs from 2010 to 2019.Number of participated patients was from 32 to 87. The population involved patients with AD. The details of characteristics of the included studies are summarized in Table 3.

**Table 3:** Basic characteristics of some of the included studies.

Study NO.	Year	First author	Journal	Country	Study design	Principle health problem	Patient	Final sample size	Duration	Intervention	Comparison	Main outcomes
1	2010	Niu, Y. X.	Clin Rehabil	China	RCT	AD	Patients were recruited from a military sanatorium in Beijing, China	32	10 wk	placebo	CST	MMSE; the Neuropsychiatric Inventory
2	2011	Förster, S.	J Alzheimers Dis	Germany	RCT	mild AD, aMCI	patients who were at the Dementia Research Section and Memory Clinic of the Alzheimer Memo-rial Center and Geriatric Psychiatry Branch	36	6 mo	Met monthly and received pencil and paper exercises for self-study	group-based cognitive intervention	MMSE; ADAS-cog
3	2017	Giovagnoli, A. R.	Neurol Sci	Italy	RCT	AD	Patients were recruited at 1 center, who were mild to moderate dementia (MMSE, score >15)	39	24 wk	AMT; NE	CT	WFT; SST; STAI; BDI
4	2017	Tsantali, E.	Brain impairment	Greece	RCT	AD	The patients were(1) recruitment of the participants from the Alzheimer's outpatients list according to the stage of the disease (mild), their history, age and education level and their MMSE score and (2) applying the other inclusion criteria (neuropsychological profile, type of medication, lifestyle).	55	12 mo	CS; CD	CT	MMSE; CAMCOG; BNT; PPT; RBMT
5	2018	Nousia, A.	Neural Plast	Greece	RCT	AD	Patients who attended the Clinical Laboratory of Speech and Language Therapy of the Technological Educational Institute of Epirus were screened for participation in the study from December of 2016 until July of 2017	50	15 wk	Receive usual standard clinical care	MCT	Delayed memory; TMT; DSF; CDT; DSB
6	2019	Cavallo, M.	J Appl Gerontol	Italy	RCT	AD	All patients received a diagnosis of early stage AD within 12 months prior the beginning of the study.	36	12 mo	Control cognitive intervention	Computerized training (CT)	MMSE; Digit span; Two-syllable words test RBM; GNT; Token test; VOSP; Verbal fluency; Hayling test; Brixton test
7	2019	Fonte, C.	Aging (Albany NY)	Italy	RCT	AD; MCI	27 MCI (11 males/16 females) and 60 patients with AD (21 males/39 females)	87	9 mo	PT; CTRL	CT	DCT;ADAS-Cog

## 4 Discussion

We will highlight the strengths and limitations during identifying evidence. In order to improve accuracy, two main researchers will complete the data extraction and risk of bias assessment independently, there are several limitations to this network meta-analysis. First, the quality of the included studies was not optimal. When evaluating these studies, we found that many lacked details on randomization or blinding. Second, although we evaluated the studies according to the tool, there were no quantitative index that can evaluate the only artificial risk of bias. Third, because we used strict inclusion and exclusion criteria, the amount of included studies was less, which may have influenced the strength of the evidence.^[27]^

We hope that this study can screen the best cognitive intervention methods. Moreover, more research on the treatment mechanism is needed to understand how these technologies work and how they work to improve. In the future, we can in-depth study the cost-effectiveness of cognitive behavioral therapy.

## Acknowledgments

We thank Dr. Jin-Hui Tian for his guidance on the methodology of this study.

## Author contributions

Ya-Qin Lu, Wan-Qiang Chen and Fang-Fang Wu conceived the study, developed the criteria, drafted the protocol and revised the manuscript.

Hong-Bo Lv and Fang-Fang Wu designed the inclusion/exclusion criteria and the searching strategy.

Wen-Ting Xing and Fang-Fang Wu searched for the literature and analyze the data.

Hong-Bo Lv, Qi Liu and Yong-Gui Ge extracted and analyzed the data.

Jun-Ping Liu designed a data extraction table and extracted the data.

All authors have read and approved the final manuscript.

**Conceptualization:** Wan-Qiang Chen, FangFang Wu, Hong-Bo Lv, Jun-Ping Liu.

**Data curation:** FangFang Wu, Hong-Bo Lv, Wen-Ting Xing, Qi Liu, Jun-Ping Liu, Yong-Gui Ge.

**Formal analysis:** Qi Liu, Yong-Gui Ge, Ya-Qin Lu.

**Methodology:** Wan-Qiang Chen, Hong-Bo Lv, Ya-Qin Lu.

**Software:** FangFang Wu, Wen-Ting Xing, Qi Liu.

**Writing – original draft:** Wan-Qiang Chen, FangFang Wu, Ya-Qin Lu.

**Writing – review & editing:** Wan-Qiang Chen, FangFang Wu, Ya-Qin Lu.
